# What Parents Think about Giving Nonnutritive Sweeteners to Their Children: A Pilot Study

**DOI:** 10.1155/2014/819872

**Published:** 2014-11-04

**Authors:** Allison C. Sylvetsky, Mitchell Greenberg, Xiongce Zhao, Kristina I. Rother

**Affiliations:** ^1^Section on Pediatric Diabetes & Metabolism, NIDDK, NIH, 9000 Rockville Pike, Building 10, Room 8C432A, Bethesda, MD 20892, USA; ^2^Department of Exercise and Nutrition Sciences, The George Washington University, 950 New Hampshire Avenue NW, Room 204, Washington, DC 20052, USA; ^3^Diabetes, Endocrinology, and Obesity Branch, NIDDK, NIH, 9000 Rockville Pike, Building 10, Room 7C432B, Bethesda, MD 20892, USA

## Abstract

*Objective.* To evaluate parental attitudes toward providing foods and beverages with nonnutritive sweeteners (NNS) to their children and to explore parental ability to recognize NNS in packaged foods and beverages. *Methods.* 120 parents of children ≥ 1 and ≤18 years of age completed brief questionnaires upon entering or exiting a grocery store. Parental attitudes toward NNS were assessed using an interviewer-assisted survey. Parental selection of packaged food and beverages (with and without NNS) was evaluated during a shopping simulation activity. Parental ability to identify products with NNS was tested with a NNS recognition test. *Results.* Most parents (72%) disagreed with the statement “NNS are safe for my child to consume.” This was not reflected during the shopping simulation activity because about one-quarter of items selected by parents contained NNS. Parents correctly identified only 23% of NNS-containing items presented as foods or beverages which were sweetened with NNS. *Conclusions.* The negative parental attitudes toward providing NNS to their children raise the question whether parents are willing to replace added sugars with NNS in an effort to reduce their child's calorie intake. Our findings also suggest that food labeling should be revised in order for consumers to more easily identify NNS in foods and beverages.

## 1. Introduction

Sugar-sweetened foods and beverages contribute to weight gain in children and adults [1]. As a result, lower calorie alternatives have become widely available and many of these products contain nonnutritive sweeteners (NNS), such as acesulfame potassium, aspartame, saccharin, and sucralose. While NNS are chemically diverse compounds, they are all sweet-tasting and contribute no or few calories. At present, it remains unclear whether substitution of caloric sugars with NNS can ameliorate weight gain [2, 3]. Many physiological and psychological explanations for the inconsistent effects on weight have been proposed [4, 5] (for a detailed review, see Pepino and Bourne 2011) and recent data has suggested that NNS may affect weight and weight-related chronic diseases through altering glycemia [6–8], satiety and food intake [9], taste preferences [10], and/or the composition of the gut microbiota [6, 11]. However, the clinical relevance of these findings in humans has not been well studied and little is known about the long-term effects of NNS consumption beginning in childhood [3].

A recent survey conducted by the International Food Information Council Foundation (IFIC) found that 20% of adult participants reported consciously avoiding NNS, though specific concerns about their use were not detailed [12]. Similarly, many consumers reported specifically avoiding NNS in the Sweetener360 study (http://www.cornnaturally.com/Sweetener-360), yet these same consumers were found to purchase foods and beverages sweetened with NNS. Given these findings in adults, the current pilot study aimed to evaluate parental attitudes toward providing NNS-containing items to their children and to explore parents' ability to identify commercially available products containing NNS.

## 2. Materials and Methods

The Office of Human Subjects Research (OHSR) at the National Institutes of Health (NIH) approved the content of the questionnaires and the procedure. The management of the national grocery chain granted permission to conduct the project on store premises. No written consent was obtained, as all data were obtained anonymously.

### 2.1. Sample

This survey-based study was conducted in a convenience sample of 125 adults recruited upon entering or exiting a grocery store in Kensington, Maryland, a suburb of Washington, DC. Given the exploratory nature of this study, a sample size calculation was not performed prior to the start of the study. Individuals were eligible to participate if they were ≥18 years of age, if they had a child aged ≥1 year and ≤18 years, and if they spoke and understood English. Volunteers were excluded if they indicated that they were employees of a food or a beverage company, nutritionists, or health policy specialists.

### 2.2. Procedure

Parents were surveyed throughout the summer of 2012 at varying times of the day and on various days of the week to avoid selection bias. Members of the study team approached parents as they entered or exited the grocery store and asked parents if “they would be willing to complete a brief interview about their family's grocery preferences?” After providing verbal informed consent, participants were asked to partake in 4 components of our study: (1) to complete an 11-item interviewer-assisted demographic questionnaire, (2) to indicate which products they would like to purchase for their families during a grocery shopping simulation activity, (3) to identify NNS in foods and beverages as part of an NNS recognition test, and (4) to indicate their agreement with statements related to NNS in their children's diet. Commercially available food and beverage items (*n* = 142) were displayed on a large table and were arranged in the same order each day. Forty-four of the 142 items contained NNS. The NNS products were selected to include a wide range of low-calorie beverages, no-calorie beverages, low-calorie condiments, low-calorie desserts, and low-calorie grains and cereals. Non-NNS-containing products (up to three per NNS-containing item) were chosen which most closely resembled an NNS-containing food or beverage. Fresh fruits, vegetables, and other perishable, nonpackaged foods were not displayed. Presentation of foods and beverages was such that the front of the package was easily visible and all items were numerically coded.

Parents were asked to indicate which items they would hypothetically purchase for their family by stating the numeric code of each item selected and were instructed to assume that price was not an issue. Next, study team members explained what was meant by the term NNS and provided examples of the chemical names of NNS (e.g., aspartame, sucralose) as well as examples of the tabletop packets that contain NNS (e.g., Equal, Splenda). Before being asked to identify NNS-containing items by their numeric codes, participants had the opportunity to ask study team members for clarification about what constituted NNS. Finally, participants completed a 28-item questionnaire to assess their attitudes toward providing foods and beverages with NNS to their child. Parents were asked to provide answers that applied to a single child (if they had more than 1 child in the home) and individuals were only eligible to complete the survey once. After completing the survey, study team members disclosed which of the items presented in the grocery shopping simulation activity contained NNS. Participants received a $20 grocery store gift card as compensation for their participation in the study.

### 2.3. Measures

#### 2.3.1. Sociodemographic Characteristics

Sociodemographic variables included gender, age group (18–25, 26–35, 36–45, 46–55, or >55 years), race and ethnicity (non-Hispanic white, non-Hispanic black, Hispanic, or other), self-reported weight and height, perceived weight category (underweight, normal weight, overweight, and obese), number of children in the household, occupation, and educational attainment (≤high school, some college, Bachelor's degree, Master's degree, and Doctorate degree). Body mass index (BMI) was calculated (weight in kg ÷ height in m^2^) using self-reported height and weight.

#### 2.3.2. Grocery Shopping Simulation Activity

The percentage of NNS-containing items out of the total number of groceries selected was calculated for each participant. For example, if a participant selected 24 items and 12 of these items contained NNS, this participant's* NNS grocery score* would be 50% (12 out of 24 × 100).

#### 2.3.3. Nonnutritive Sweetener Recognition Test

An* NNS recognition score* was calculated based on how many of the 44 NNS-containing items were identified out of the 142 presented products. For example, if a participant correctly identified 15 NNS-containing items, this participant's NNS recognition score would be 34% (15 out of 44 × 100).

#### 2.3.4. Nonnutritive Sweetener Attitudes

NNS attitudes were measured using a 28-item questionnaire. The wording and items in the NNS-attitude questionnaire were developed based upon the expertise of the study team. Members of the study team read the statements out loud and parents indicated their level of agreement with each statement using a 5-point Likert scale, where “1” was strongly disagree and “5” was strongly agree.

### 2.4. Statistical Analysis

Descriptive statistics were calculated for NNS-grocery and NNS-recognition scores. *P* values were calculated using* t*-tests, ANOVA, and chi-square test as appropriate. A *P* value < 0.05 was considered statistically significant.

## 3. Results

The sociodemographic characteristics of our sample are shown in Table 1. Of approximately 450 parents approached, 125 (28%) agreed to participate in the study, and 120 were included in the analyses. Two individuals were excluded because their child was less than 1 year old, while three parents were excluded due to lack of compliance with study procedures. Seventy-eight percent of participants were female and most self-identified as either non-Hispanic white (44%) or non-Hispanic black (34%). Fifty-one percent of participants perceived themselves as having a normal weight, and the mean parental BMI (based on self-reported weight and height) was 26.4 ± 5.5 kg/m^2^.

### 3.1. Grocery Choices

The average number of groceries that the participants indicated they would hypothetically buy was 22 items (16%) out of the 142 packaged foods and beverages presented. On average, 22% of the groceries selected by parents contained NNS. Parent BMI was not a significant predictor of the number of groceries selected.

### 3.2. NNS Recognition

**Figure 1:**
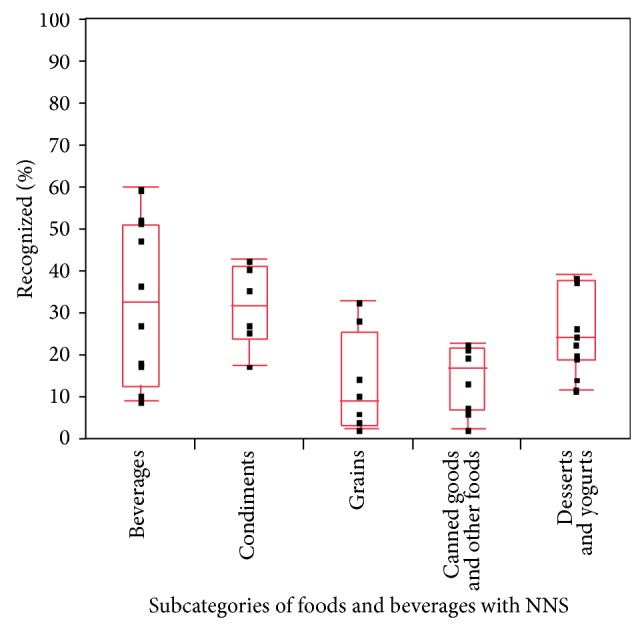
Parental ability to recognize foods and beverages (*n* = 44) containing nonnutritive sweeteners. Recognition of NNS varied based on the type of food and beverage presented (*P* = 0.02). Participants generally recognized NNS with higher frequency in beverages, condiments, desserts, and yogurts, while NNS in grains, canned goods, and other foods were more frequently overlooked. Each black dot corresponds to individual food or beverage items within each category.

The mean NNS-recognition score was 23 ± 14%. As shown in Figure 1, recognition of NNS depended on the type of food and beverage presented (*P* = 0.02). Participants generally recognized NNS with higher frequency in beverages, condiments, desserts, and yogurts while NNS in grains, canned goods, and other foods were more frequently overlooked. NNS recognition was inversely associated with BMI (*P* < 0.002).

### 3.3. Nonnutritive Sweetener Attitudes

Parental agreement with survey items assessing NNS acceptance is shown in Table 2. Seventy-two percent of parents disagreed with the statement “NNS are safe for my child to use,” while an additional 12% neither agreed nor disagreed. Fifty-eight percent of participants indicated that they looked for NNS in the ingredients lists on foods and beverages because they wanted to avoid purchasing items that contained NNS. These parents were significantly more accurate in recognizing foods and beverages with NNS (*P* = 0.003). However, the mean NNS-grocery score did not differ between parents who reported avoiding foods and beverages with NNS and those who did not.

Parents indicated a preference for items labeled “reduced sugar” and “no sugar added” (53% and 52%, resp.). Fewer indicated that they sought out items labeled “light,” “low carb,” or “sugar-free” (37%, 33%, and 22%, resp.). Definitions of relevant food claims are available on the US Food and Drug Administration (FDA) website [13].

## 4. Discussion

The findings of the current pilot study indicate that the majority of parents do not believe that NNS are safe for their children to consume. We found that parental recognition of NNS in commercially available foods and beverages was limited, as parents were unable to identify NNS in 77% of the NNS-containing products presented. The negative parental views observed in this pilot study raise the question as to whether parents are willing to replace sources of added sugars in children's diets with foods and beverages containing NNS.

Over half of parents reported a preference for foods and beverages with “no sugar added” nutrient content claims. These items frequently contain NNS. Meanwhile, the “sugar-free” and “light” nutrient content claims, which may more obviously convey the replacement of caloric sugars with NNS, were perceived less favorably. This suggests that certain food label claims (e.g., “no sugar added”) inadvertently encourage parents to select grocery items with NNS. This discrepancy between reported parental attitudes toward NNS and their selection of items that contain them draws attention to issues in parental nutrition literacy.

Our findings are particularly important because the consumption of NNS has increased in the United States over the last decade [14] and NNS consumption is likely to continue to increase. For example, school beverage guidelines [15], taxation of sugar-sweetened beverages [16], and alterations to the food label to highlight added sugar content [17] have been proposed, which may encourage sugar-sweetened beverage consumers to switch to NNS-containing beverages. While NNS have the potential to serve as a valuable dietary tool in reducing caloric intake from added sugars, their safety remains a topic of debate and conclusive evidence of their safety for children specifically is lacking. Epidemiological studies support an association between NNS, weight gain, and other cardiometabolic abnormalities, but prospective, interventional studies are inconclusive. Two recent clinical trials in children and adolescents have shown less weight gain upon replacing sugar-sweetened sodas with NNS sweetened beverages [18, 19], but these trials did not include a control arm with unsweetened beverages (e.g., water). It is therefore necessary to elucidate the long-term metabolic and health effects of NNS in order to appropriately educate parents about the use of NNS among children.

Limitations of our study include a relatively small convenience sample at a single grocery store. While the use of a diverse population of parents representing various race/ethnicity groups added strength to our study, the high level of educational attainment in our sample challenges the generalizability of our findings to the general population. However, given that parental ability to recognize NNS-containing foods and beverages was low despite the high level of educational attainment, we expect that recognition of NNS would be even lower among less educated parents. We also used nonvalidated survey instruments and activities and did not collect qualitative data or information about the parents' habitual NNS consumption habits to further explain our findings. Our participants were required to choose products in a setting which simulated grocery shopping in a free-living population.

## 5. Conclusions

Our study clearly demonstrates that parents generally do not perceive NNS as safe for their children and frequently do not recognize common NNS-containing foods and beverages. Our findings support the need to identify and implement simple approaches to food labeling in order to facilitate informed dietary choices among consumers and to improve parental knowledge about foods and beverage composition. Considering the recently proposed changes in food labeling [17] and heightened consumer awareness about the health effects of added sugars [20], parents may increasingly seek foods with lower added sugar content, while failing to recognize that many of these foods and beverages will instead contain NNS.

The results of this study also challenge the extent to which parents may be willing to provide their children with NNS and emphasize the need to identify other strategies to reduce added sugar intake and mitigate excessive weight gain in children. The low parental recognition of NNS and strikingly negative parental attitudes towards consumption of NNS-containing foods and beverages should also be considered by pediatricians and other practitioners when counseling parents to reduce their child's sugar intake. Furthermore, our findings call attention to the need to elucidate the long-term health effects of NNS in order to confirm their safety and appropriately educate both clinicians and parents.

## Figures and Tables

**Table 1:** Sociodemographic characteristics of sample based on NNS recognition (*n* = 120).

	*N* (% of total)
All participants	
*n*	120 (100%)
Gender	
Male	26 (22%)
Female	94 (78%)
Age group	
18–25	8 (7%)
26–35	23 (19%)
36–45	44 (37%)
46–55	34 (28%)
55+	11 (9%)
Race	
Non-Hispanic white	53 (44%)
Non-Hispanic black	41 (34%)
Hispanic	18 (15%)
Other	8 (6%)
BMI (kg/m^2^)^a^	26.4 ± 5.5
Education	
≤High school	20 (17%)
Some college	21 (18%)
Bachelor's	39 (33%)
Master's/Doctorate	40 (33%)

^a^BMI was calculated based on self-reported height and weight.

**Table 2:** Percent agreement with questionnaire items related to NNS, sugar-related nutrient content claims, and parental concern regarding specific macronutrients.

Statement	Percent agreement (%)
I seek out items labeled “reduced sugar”	53
I seek out items labeled “no sugar added”	52
I seek out items labeled light	37
I seek out items labeled low carb	33
I seek out items labeled sugar-free	22
I read the ingredients in the packaged items that I purchase	64
I look for NNS in packaged foods and beverages because I want to avoid them	58
I am concerned with the calorie content of the items that I select	52
I am concerned with the sugar content of the items that I select	73
I am concerned with the fat content of the items that I select	68
Nonnutritive sweeteners (i.e., Splenda, Sweet N Low, and Equal) are safe for my child to use	16
I recommend that my child use diet (NNS) foods and beverages because I am concerned about his/her sugar intake	14
I recommend that my child use diet (NNS) foods and beverages because I am concerned about his/her weight	13
